# Patients with Chronically Diseased Livers Have Lower Incidence of Colorectal Liver Metastases: A Meta-Analysis

**DOI:** 10.1371/journal.pone.0108618

**Published:** 2014-09-29

**Authors:** Bin Cai, Kai Liao, Xian-qing Song, Wei-yuan Wei, Yuan Zhuang, Sen Zhang

**Affiliations:** 1 Department of Colorectal Surgery, First Affiliated Hospital of Guangxi Medical University, Nanning, Guangxi, People's Republic of China; 2 Department of Gastrointestinal Surgery, First Affiliated Hospital of Guangxi Medical University, Nanning, Guangxi, People's Republic of China; 3 School of preclinical Medicine, Guangxi Medical University, Nanning, Guangxi, People's Republic of China; University of Pisa, Italy

## Abstract

**Background:**

70 years ago, it was put forward that the diseased liver was not a favorable soil for metastatic tumor cells. In addition, a few studies have demonstrated that rare occurrence of colorectal liver metastases among patients with fatty liver, cirrhosis or chronic hepatitis B and C virus infection. We performed a meta-analysis to verify the association between the incidences of colorectal liver metastases with chronically diseased livers.

**Methods:**

Relevant studies were identified by a search of electronic database PubMed, Cochrane Library, OVID, Web of Science and CNKI (up to February 24, 2014). Pooled odds ratio (OR) with 95% confidence interval (CI) was calculated using random- or fixed-effect models when appropriate. Meta-analysis and publication bias (Bgger's test) was evaluated with STATA 12.0.

**Results:**

A total of 10,349 colorectal cancer patients from 10 studies were included. The meta-analysis result showed there was a significant difference in the incidences of colorectal liver metastases between patients with normal and chronically diseased livers (OR = 0.32; 95% CI 95%: 0.26–0.38, *P* = 0.000 fixed-effects model). The result of Begg's test (Pr>|z| = 0.089; *P*>0.05) revealed no publication bias.

**Conclusions:**

The results of this meta-analysis demonstrated that patients with chronically diseased livers had significantly lower incidences of colorectal liver metastases than those with normal livers.

## Introduction

Colorectal cancer (CRC) is a high morbidity malignancy tumor over the world, which is predicted will account for 8% of new cancer cases in the United States in 2014 [1]. Tumor recurrence and distant metastasis are the main causes of death in CRCs [2]. The liver is the most common site for metastatic colorectal cancer. It was reported that approximately 40% of advanced colorectal cancer patients developed liver metastases [3], [4]. In 1942, Lisa et al [5] reported that the cirrhotic liver was not a favorable soil for metastatic tumor cells. They found that, in 782 autopsy cases with malignant tumors, there were only 6 cases with metastatic cancer in cirrhotic liver. A theory for this phenomenon was that an inappropriate environment for the transplanted tumor cells was formed by the diseased liver; this meant the “soil” was not favorable for the “seed” to grow [5].

During the past twenty years, several studies have discovered a low incidence of colorectal liver metastases in patients with liver diseases, including fatty liver [6], cirrhosis [7] and chronic hepatitis B and C virus infection [8]. However, the precise reason of the above findings is still unclear. Since only few articles reported this issue, we collected all relevant articles and carried out a meta-analysis to compare the incidences of colorectal liver metastases in normal and chronically diseased livers.

## Methods

### Literature collection

We collected the potentially relevant studies through a search in electronic database PubMed, Cochrane Library, OVID, Web of Science and Chinese National Knowledge Infrastructure (CNKI). Chronically diseased liver included cirrhosis, fatty liver and hepatitis virus infection. The keywords used for the search including “colorectal cancer”, “diseased liver”, “hepatitis”, “fatty liver”, “cirrhosis” and “liver metastasis”. All non-English articles were translated in English and then analyzed. The latest search was updated on February 24, 2014.

### Inclusion and exclusion criteria

Inclusion criteria: (1) studies evaluating the association between colorectal liver metastases and chronically diseased livers; (2) only patients with advanced colorectal cancer were included in the study, because early-staged colorectal cancers such as a lesion confined to the mucosa or submucosa rarely metastasized to the liver; (3) all patients were closely followed up; (4) containing useful figures including numbers of the two groups (diseased liver group and normal liver group) and numbers of liver metastases in each group. Exclusion criteria: (1) animal studies, pharmaceutical researches, case reports; (2) reports without usable data; (3) duplicate publications.

### Date extraction

Two investigators (CB and LK) extracted data from eligible studies independently, according to the inclusion and exclusion criteria above. For disagreements, a consensus was reached by a third investigator (ZS). The following information was collected from each study: first author, publication date, country of origin, ethnicity, type of liver diseases, number of each group (diseased liver group and normal liver group), and number of liver metastases in each group.

### Statistical methods

Meta-analysis was performed by using Stata 12.0 software (Stata Corporation, College Station, TX, USA). To determine the statistical heterogeneity of the studies, Chi-square-based Q test and *I^2^* statistics were used. For the Q test, A *P* value less than 0.05 indicated significant heterogeneity; for the *I^2^* statistics, an *I^2^* value greater than 50% was considered severe heterogeneity. The potential publication bias was assessed using a “funnel plot” and the Begg's test. The fixed-effects model was adopted in the initial calculation of odds ratio with corresponding 95% CIs. If there was a significant statistical heterogeneity among the studies, the random-effects model was applied for the analysis. By comparing the incidences of colorectal liver metastases in normal and chronically diseased livers, we tried to explore the impact of liver diseases on colorectal liver metastases.

## Results

### Studies characteristics

**Figure 1:**
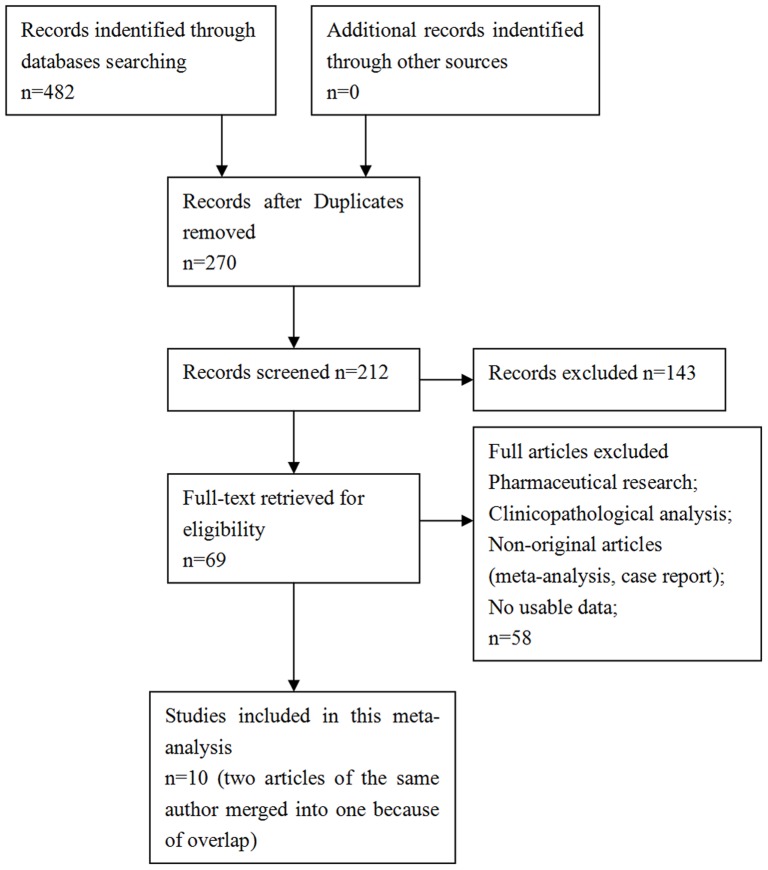
Flowchart presenting the steps of literature search and selection.

A total of 10 retrospective studies published between 1992 and 2013 were identified as eligible according to the selection criteria **Figure 1**. Five studies evaluated patients from China, four evaluated patients from Japan and one evaluated patients from Italy [6]–[16]. These studies included a total of 10,349 patients. The overall incidence of colorectal liver metastases in diseased liver groups was 8.92% (138/1547), and in normal liver groups it was 21.445% (1887/8802). All the diagnoses of colorectal cancer relied on pathology, all the judgements of synchronous or metachronous liver metastases were confirmed by biopsy or diagnostic imaging (CT, MRI or PET-CT). All the diagnoses of hepatitis infection were based on serological tests of hepatitis virus, including HBsAg, anti-HBs, HBeAg, anti-HBe, and anti-HBc. The patients who had one or more items positive in the hepatitis tests were considered as the chronic hepatitis virus infection group. The diagnoses of liver cirrhosis had no unified standard. Some were according to the assessment of a combination of laboratory parameters (serum bilirubin, serum albumin, prothrombin time international normalized ratio) and clinical parameters (encephalopathy and ascites). Some were based on histological diagnosis or ultrasound/CT scan. The diagnoses of fatty liver were based on ultrasound/CT scan. The main characteristics were summarized in **Table 1**.

**Table 1:** Main characteristics of the eligible studies.

First author	Year	country	Ethnicity	types of liver diseases	Total number	Diseased liver	Diseased liver with liver metastases	Non-diseased liver	Non-diseased liver with livermetastases
Uetsuji	1992	Japan	Asian	Cirrhosis	250	46	0	204	40
Hayashi	1997	Japan	Asian	Fatty liver	839	121	2	718	115
Utsunomiya	1999	Japan	Asian	Hepatitis infection	438	37	3	401	85
Song	2001	China	Asian	Hepatitis infection	512	74	10	438	119
Iasome	2005	Italy	Caucasian	Hepatitis infection/cirrhosis/fatty liver	834	291	28	543	174
Qian	2010	China	Asian	Hepatitis infection/cirrhosis	2352	140	11	2212	517
Qiu	2011	China	Asian	Hepatitis infection	1298	332	47	966	272
Wang	2012	China	Asian	Hepatitis infection	354	70	2	284	48
Zeng	2013	China	Asian	Hepatitis infection	2868	373	33	2495	465
Murono	2013	Japan	Asian	Fatty liver	604	63	2	541	52

### Meta-analysis Results

**Figure 2:**
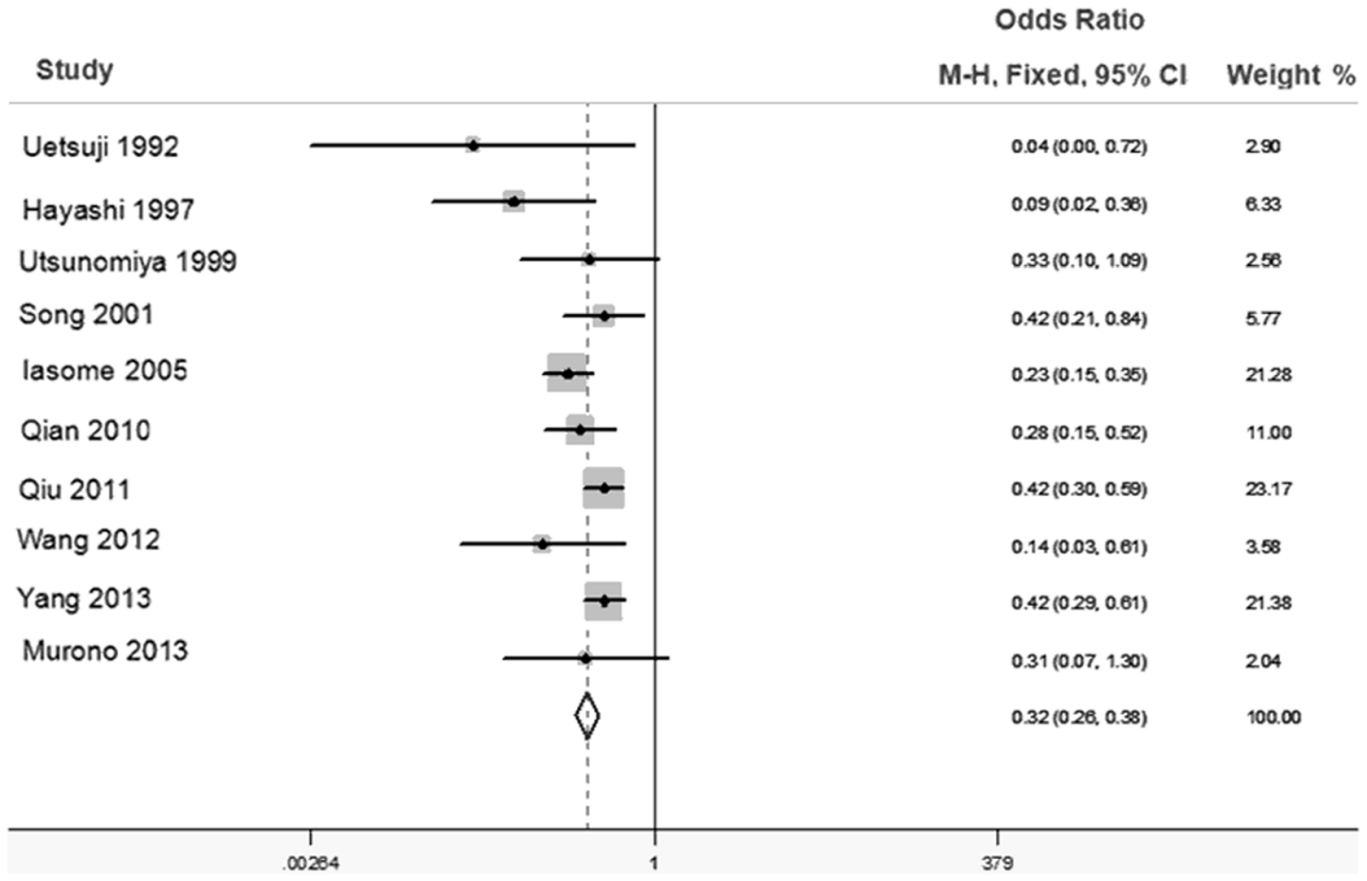
Forest plot for the association between incidences of colorectal liver metastases with chronically diseased liver. Forest plot of OR was assessed for the differences of incidence of colorectal liver metastases between the diseased liver group and normal liver group (OR = 0.32, 95%CI = 0.26–0.38 fixed effects model).

As **Figure 2** showed there was a moderate heterogeneity among the trials (*I^2^* = 37.4% *P*>0.05), and then the fixed-effects model was adopted. The odds ratio, expressed as diseased liver group versus normal liver group, was 0.32 (95% CI: 0.26–0.38, *P* = 0.000 fixed-effects model). Through comparing the incidences of colorectal liver metastases between the diseased liver group and normal liver group, we found there was a significant difference in the incidences of colorectal liver metastases between the two groups. This result demonstrated that patients with chronically diseased livers had significantly lower incidences of colorectal liver metastases than those with normal livers.

### Publication bias

**Figure 3:**
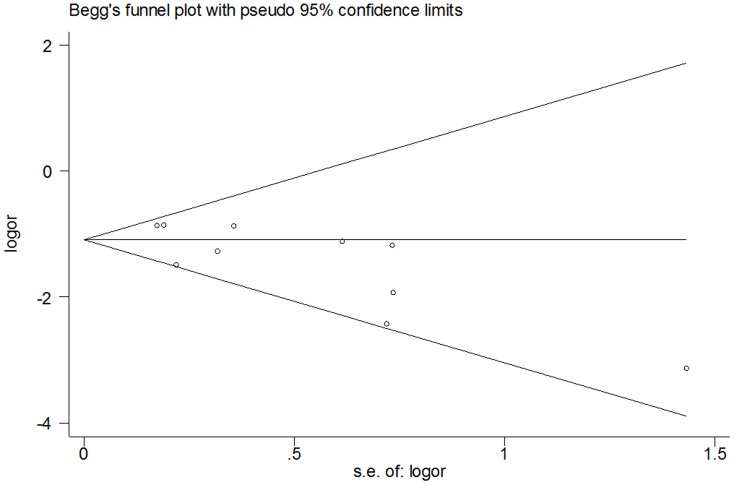
Funnel plot analysis of potential publication bias.

**Figure 3** showed the Begg's funnel plot with pseudo 95% CIs. No significant publication bias was observed. Bias was assessed statistically using the Begg's test; still, the results of Begg's test revealed no publication bias (Pr>|z| = 0.089; *P*>0.05).

## Discussion

This meta-analysis showed that colorectal cancer patients with chronically diseased livers had significantly lower incidences of colorectal liver metastases than those with normal livers. The reason for this phenomenon is still unknown. Studies have shown that clinicopathological characteristics of primary colorectal cancers were not altered by viral hepatitis [8], [13], suggesting that the different incidences of colorectal liver metastases between the diseased liver group and normal liver group were not caused by variation of pathological factors in primary cancers.

Karube [17] found the pyrimidine nucleoside phosphorylase (PyNPase) activity and the microvessel density in the metastatic liver lesion in fatty liver rats were significantly lower than non-fatty liver rats. They suggested that the decreased activity of PyNPase and neovascularization in the metastatic lesion were closely related to fewer liver metastases. In addition, evidences showed that disorders of fat metabolism, such as increased omega-3 fatty acids and medium-chain triglycerides in the liver cells inhibited tumor cell proliferation and angiogenesis, which could prevent the growth of tumor cells [18].

Cirrhosis, as defined by the World Health Organization (WHO), was “a diffuse process characterized by fibrosis and conversion of normal liver architecture into structurally abnormal nodules” [19]. In cirrhotic livers, Kupffer cells were activated and released constitutive amount of proinflammatory factors, such as tumor necrosis factor-α (TNF-α) and interleukin −l (IL-l). Song et al [20] investigated the influence of activated Kupffer cells from cirrhotic rat livers on hepatic colonization and FasR-mediated apoptosis of colon cancer cells. They found Kupffer cells in cirrhotic livers sensitized metastatic colon cancer cells to FasR mediated apoptosis by up-regulating their expression of Fas receptors, which thus prepared the malignancies to be eliminated by tumour-infiltrating lymphocytes. Hence, activation of Kupffer cells during hepatic cirrhosis on one hand resulted in tissue damage and fibrogenesis in livers, but on the other hand inhibited the hepatic matastasis formation of colon cancers.

Seitz [21] reported that high metalloproteinase inhibitor contents and especially altered lectins or lectin binding sites in cirrhosis of the liver might help to explain the rare event “metastasis in cirrhosis”. Pathophysiological pathway of cirrhosis underwent through the process of extracellular matrix remodeling leading to new collagen formation and deposition [22]. Matrix metalloproteinases (MMPs) and their tissue inhibitors (TIMPs) played important roles in the process of matrix degrading and remodeling. In the process of fibrosis, the overall MMP activity decreased, due to increased expression of TIMPs and other anti-proteases expressed by hepatic stellate cells and hepatocytes. Therefore, increased expression of TIMPs may have inhibitory role in the process of colonization and formation of colorectal metastasis in chronically injured liver. In addition, cirrhosis was associated with increased intrahepatic resistance to portal flow. Mittal et al [23] demonstrated that there was a significant fall in peak venous velocity (PVV) with the increasing severity of the grade of cirrhosis. Moreover, a reversed flow in the portal venous system was observed in cirrhotic patients. These hemodynamic events were responsible for the progressive fall in the portal venous velocity with an increasing severity of the portal hypertension. The disruption of portal blood flow with venovenous shunting may prevent tumour cells reaching the liver.

It has been reported that hepatitis virus infection resulted in a high state of the immune response in livers [24]. Cytotoxic T lymphocytes (CTL) and Kupffer cells were main components of the immune response during HBV infection. HBV replication activated the specific lytic pathways of cell injury by CTL and Kupffer cells [25], which effectively killed metastatic cancer cells just after lodging in the sinusoids [26], [27]. This coincides with the result reported by Song et al [13] that HBV infection with viral replication, which was determined by the presence of HBeAg and HBV DNA in serum, reduced the incidence of colorectal liver metastases in CRCs; whereas, occurrence of liver metastases in patients with nonreplicative HBV infection was close to those without HBV infection. Moreover, HBV replication promoted immune cells to secrete tumor necrosis factor a (TNF-a) [28], which also killed metastatic cancer cells. In addition, Wang et al [29] found that HBV replication resulted in elevated protein levels of Polo-like kinase1 (Plk1) and down-regulation of SUZ12 (suppressor of zeste 12). Plk1 was found overexpressed in a variety of human tumors and its expression was associated with cellular proliferation and prognosis of tumor patients. Deregulation of Plk1 activity contributed to genetic instability, which in turn leaded to oncogenic transformation [30]. SUZ12 could combine EZH2 (enhancer of zeste homolog 2) and EED (embryonic ectoderm development), and formed polycomb repressive complex (PRC2). PRC2 participated in epigenetic silence of several tumor suppressor genes by catalyzing the trimethylation of histone H3 at lysine 27, which served as a docking site for DNA methyltransferases and histone deacetylases [31]. Recent studies implied that PRC2 and its subunits (SUZ12 and EZHZ) were often deregulated in various cancer types and their overexpressions were closely associated with carcinogenesis [32], [33].

### Conclusion

The phenomenon of the rare occurrence of metastatic colorectal cancers in chronically diseased livers has been observed for more than 70 years, but few related articles had been reported. This meta-analysis collected all related reports so far, and verified that patients with diseased livers have significantly lower incidences of colorectal liver metastases than those with normal livers. Nevertheless, it is still necessary to conduct larger size and better design studies to confirm our results. Moreover, of the ten included studies, nine studies evaluated patients from Asia. Because of this, our finding may just represent cancer patients from Asia. In addition, we suggest particular attention should be given to the precise mechanism of this phenomenon, so as to provide a research strategy on basic research and clinical prevention of colorectal liver metastases.

## Supporting Information

Checklist S1 **PRISMA Checklist.**
(PDF)
